# MicroRNA-181a is involved in the regulation of human endometrial stromal cell decidualization by inhibiting Krüppel-like factor 12

**DOI:** 10.1186/s12958-015-0019-y

**Published:** 2015-03-26

**Authors:** Qun Zhang, Hui Zhang, Yue Jiang, Bai Xue, Zhenyu Diao, Lijun Ding, Xin Zhen, Haixiang Sun, Guijun Yan, Yali Hu

**Affiliations:** Reproductive Medicine Center, Department of Obstetrics and Gynecology, Nanjing Drum Tower Hospital, Nanjing University Medical School, Nanjing, 210008 Jiangsu China; Reproductive Medicine Center, Drum Tower Clinic Medical College of Nanjing Medical University, Nanjing, 210029 Jiangsu China

**Keywords:** MicroRNA-181a, KLF12, Human endometrial stromal cell, Decidualization

## Abstract

**Background:**

The transformation of endometrium into decidua is essential for normal implantation of the blastocyst. However, the post-transcriptional regulation and the miRNAs involved in decidualization remain poorly understood. Here, we examined microRNA-181a (miR-181a) expression in decidualized human endometrial stromal cell (hESC). In addition, we investigated the functional effect of miR-181a on hESC decidualization *in vitro*.

**Methods:**

Quantitative real-time PCR (qRT-PCR) was used to detect the profile of miR-181a in decidualized hESC. qRT-PCR, enzyme-linked fluorescent assay, and immunofluorescence assay were performed to investigate decidualization marker genes’ expression after enhancing or inhibition of miR-181a expression in hESC. Luciferase reporter assay, western blotting, qRT-PCR, and immunofluorescence assay were carried out to identify the relationship between miR-181a and Krüppel-like factor 12 (KLF12).

**Results:**

miR-181a expression levels increased dramatically in hESC treated with 8-Br-cAMP and MPA. Increased miR-181a expression promoted hESC decidualization-related gene expression and morphological transformation; conversely, inhibition of miR-181a expression compromised hESC decidualization *in vitro*. Further analysis confirmed that miR-181a interacted with the 3′ untranslated region of the transcription factor KLF12 and down-regulated KLF12 at the transcriptional and translational levels. KLF12 overexpression abolished miR-181a-induced decidualization.

**Conclusions:**

Our findings suggest that miR-181a plays a functionally important role in human endometrial stromal cell decidualization *in vitro* by inhibiting KLF12.

## Background

Decidualization of the endometrial stroma is a precondition for the successful establishment of pregnancy. In humans, this process is initiated in the mid-secretory phase of the menstrual cycle and is triggered by ovarian sex steroid hormones independent of pregnancy [1,2]. The decidual reaction consists of a dramatic morphological and biochemical transformation of the endometrial stroma in which the stromal fibroblasts differentiate to become rounded, relatively large epithelioid-like or polygonal, secretory decidual cells [3]. Human decidual cells produce specific molecules, such as regulatory factors (prolactin (PRL) and insulin-like growth factor binding protein-1 (IGFBP-1)), inflammatory mediators (IL-1, IL-6, IL-8, and TNF-*α*), and specific extracellular matrix proteins (laminin, type IV collagen, and fibronectin) [4-7]. PRL and IGFBP-1 levels are generally used as biochemical decidualization markers of progestin-induced human endometrial stromal cell (hESC) differentiation. A number of transcription factors and autocrine/paracrine factors have been identified that cooperatively control the decidualization process. However, little information is available regarding the post-transcriptional regulation of this process.

MicroRNAs (miRNAs) have emerged as key post-transcriptional regulators. An estimated 30–50% of protein-coding genes serve as potential miRNA targets. miRNAs regulate and influence a variety of cellular activities, including cell growth, differentiation, apoptosis, and metabolism [8]. miRNAs are small (approximately 20–22 nt), noncoding RNAs that generally base-pair within the 3′ untranslated region (3′UTR) of target mRNAs, causing translational inhibition and/or mRNA degradation [9]. Recently, the conditional inactivation of Dicer has provided evidence for the pivotal functions of miRNAs in ovarian as well as oviductal and uterine stromal cell development [10]. Dicer expression increases and is a requirement during human endometrial stromal decidualization *in vitro* [11]. The aberrant expression of some miRNAs has been correlated with various endometrial diseases, such as endometriosis, repeated implantation failure (RIF), and endometrial cancer [12-14].

MicroRNA-181a (miR-181a), which belongs to the miR-181 family, is a key modulator of cellular differentiation. Based on microRNA microarray analysis, L. Su et al. found high miR-181a/c expression on day 15 of gestation, followed by decreased expression on gestational days 26 and 50 in the porcine endometrium during pregnancy [15]. Various potential miR-181 family targets, such as ETS1, CREB1/3, Esr1, and PGR, are involved during differentiation and decidualization events [16-18]. The Krüppel-like factor (KLF) family members are revealed to play critical roles in regulating the process of embryo implantation, such as KLF9 and KLF13 [19,20]. KLF12, another member of KLF family, binds to the CAGTGGG sequence within target gene promoter regions and represses target gene expression through an N-terminal PVDLS sequence (Pro-Xaa-Asp-Leu-Ser) that promotes a physical interaction with the co-repressor CtBPs [21]. We previously demonstrated that KLF12, suppressed by 8-Br-cAMP and MPA, negatively regulates hESC decidualization by inhibiting PRL and IGFBP-1 expression [22]. In this study, we demonstrated that miR-181a was involved in the regulation of hESC decidualization by suppressing KLF12, whereas KLF12 overexpression inhibited miR-181a-mediated increases in decidualization-related gene expression and the morphological transformation of hESC, indicating that miR-181a may play an important role in human endometrial decidualization.

## Methods

### Isolation and *in vitro* decidualization of hESC

This study was approved by the Institutional Review Board of the Drum Tower Hospital of Nanjing University on December 5, 2013 (2013-081-01). This study was conducted in the Drum Tower Hospital from February 2014 to September 2014. Patient consent was received before biopsy. hESC were isolated from the mid-secretory phase of endometrial tissue of women with a normal menstrual cycle by endometrial biopsy. hESC were isolated and cultured as previously described [23].

To induce decidualization, hESC were cultured in phenol red-free DMEM/F12 medium (HyClone, Thermo Scientific, South Logan, UT, USA) containing 2.5% charcoal/dextran-treated fetal bovine serum (FBS, HyClone, Thermo Scientific, South Logan, UT, USA), 100 IU/ml penicillin, and 100 μg/ml of streptomycin supplemented with 0.5 mM 8-Br-cAMP and 1 μM MPA (Sigma, St. Louis, MO, USA) for 3 days, 6 days, or 9 days. Differentiation was assessed by examination of cell morphology under phase contrast microscopy at various times during the treatment and also by measuring the expression of decidualization-specific marker gene, namely PRL.

### Construction of adenovirus

To overexpress KLF12 and miR-181a in hESC, adenovirus vectors harboring the full-length KLF12 (Ad-KLF12) and precursor miR-181a (Ad-miR-181a) were generated using the AdMax (Microbix Biosystems, Inc., Toronto, Canada) and pSilencer™ adeno 1.0-CMV (Ambion, Austin, TX, USA) systems as previously described [22,24]. The primers for full-length KLF12 amplification were: 5′-TCTCGAATTCAATGAATATCCATATGAAGAG-3′ and 5′-TATAGGATCCTCACACCAACATATGCCTCC-3′; for precursor miR-181a amplification were: 5′-CGCGCTCGAGATACAATGTGATGTGGAGGTT-3′ and 5′-GCGCGATATCGGCCACAGTTGCATTCATTGT-3′. An adenovirus bearing LacZ (Ad-LacZ) was obtained from Clontech (Palo Alto, CA, USA) and used as the control of adenovirus infection experiments. The viruses were packaged and amplified in HEK293A cells and purified using CsCl banding followed by dialysis against 10 mM Tris-buffered saline with 10% glycerol. The viral titer was determined using HEK293A cells and the Adeno-X Rapid Titer kit (Clontech). hESC were infected with Ad-miR-181a at the 100 MOI and/or with Ad-KLF12 at the 20 MOI.

### miRNA inhibitor transfection

miR-181a inhibitor (2′-O-methyl modified oligonucleotides: 5′-mAmCmUmCmAmCmCmGmAmCmAmGmCmGmUmUmGmAmAmUmGmUmU-3′) or miRNA inhibitor negative control (miRNA inhibitor control) were synthesized by Ribobio (Guangzhou, China). miRNA inhibitor control shares no homologous region with the human genome sequences. For loss of function experiments, hESC were transfected with 100 nM of miR-181a inhibitor or of miRNA inhibitor control using Lipofectamine 2000 (Life Technologies, New York, USA).

### RNA isolation and quantitative real-time PCR (qRT-PCR)

Total RNA was extracted from hESC using Trizol reagent (Invitrogen, Carlsbad, CA, USA). Reverse transcription was performed using random primers or specific miRNA stem-loop primers, and qRT-PCR was performed on a MyiQ Single-Color Real-Time PCR Detection System (BIO-RAD, Hercules, CA, USA). To detect miR-181a expression, we used the following primers: forward, 5′-ACACTCCAGCTGGGAACATTCAACGCTGTCG-3′; reverse, 5′-GGTGTCGTGGAGTCGGCAATTCAGTTGAG-3′. The small nuclear RNA U6 was used as an internal control and was amplified with the following primers: forward, 5′-CTCGCTTCGGCAGCACA-3′; reverse, 5′-AACGCTTCACGAATTTGCGT-3′. The following primers were also used for the indicated genes: FOXO1A, 5′-CCTCTGGATTGAGCATCCAC-3′ and 5′-ATGTATGGAGGTGGGTCAGC-3′; PRL, 5′-CACTACATCCATAACCTCTC-3′ and 5′-ATGCTGACTATCAAGCTCAG-3′; IGFBP1, 5′-TATGATGGCTCGAAGGCTCTC-3′ and 5′-GTAGACGCACCAGCAGAGTC-3′; DCN, 5′-AGCTCTCCTACATCCGCATT-3′ and 5′-GCTAGCTGCATCAACTCTGC-3′; TIMP3, 5′-TGACAGGTCGCGTCTATGAT-3′ and 5′-CAACCCAGGTGATACCGATAG-3′; KLF12, 5′-CCTTTCCATAGCCAGAGCAG-3′ and 5′-TTGCATCCCTCAAAATCACA-3′; 18S rRNA, 5′-CGGCTACCACATCCAAGGAA-3′ and 5′-CTGGAATTACCGCGGCT-3′. Samples were run in duplicate with RNA preparations from three independent experiments. The fold change in expression of each gene was calculated using the 2^-△△CT^ method, and 18S rRNA or U6 served as an internal control.

### Western blotting

Briefly, protein extracts were prepared from hESC as previously described [23]. Equal amounts of total protein (30 μg) were separated on a 10% SDS-polyacrylamide gel and transferred onto polyvinylidene fluoride membranes (Millipore, Billerica, MA, USA). Immunoblotting was performed with primary antibodies against KLF12 (1:500; Santa Cruz Biotechnology, Santa Cruz, CA, USA) or β-actin (1:10,000; Abcam, Cambridge, MA, USA) followed by a goat anti-rabbit HRP-conjugated secondary antibody (1:10,000; Bioworld Technology, St. Louis Park, MN, USA). Bands were detected using an enhanced chemiluminescence kit (Amersham Biosciences Corp., Piscataway, NJ, USA).

### Luciferase reporter assay

The sequence (5′-CTGCGTATAAGGGACTGAATGTGAGGTAACTCTTATG-3′) in the 3′UTR of the human KLF12 gene containing the miR-181a seed sequence TGAATGT (pmirGLO-KLF12 3′UTR) or the sequence (CTGCGTATAAGGGAC GAGGTAACTCTTATG) that lacks TGAATGT (pmirGLO-KLF12 3′UTR mut) were subcloned in the pmirGLO Dual-Luciferase miRNA Target Expression Vector (pmirGLO vector, Promega, Madison, WI, USA). Preconfluent (70%) hESC in six-well plates were infected with Ad-miR-181a and then transfected with 300 ng of the luciferase reporter plasmids using Lipofectamine 2000 for 48 h. The cell lysates were assayed for luciferase activity using the Luciferase Assay System (Promega, Madison, WI, USA), and the activity was measured using a luminescence counter (Centro XS3 LB 960, Berthold Technologies).

### Immunofluorescence staining for F-actin filaments

hESC were grown in 8-well chambers (Millipore, Billerica, MA, USA) and fixed with 4% paraformaldehyde for 30 min at room temperature, permeabilized with 0.5% Triton X-100 in PBS, and incubated with Alexa Fluor 594-conjugated phalloidin for F-actin filaments staining (Sigma) at 4°C overnight. The cell nuclei were stained with DAPI (5 μg/mL) on the following day. Finally, images were visualized using a fluorescence microscope (Leica, Wetzlar, Germany).

### Prolactin examination by enzyme-linked fluorescent assay (ELFA)

Prolactin levels in the supernatant of hESC cultured with phenol red-free DMEM/F12 medium containing 2.5% charcoal/dextran-treated FBS were measured using the Mini-Vidas V.B. 02.96 system with Vidas prolactin kits (bioMérieux, France). The limit of detection of this kit was 0.5 ng/mL.

### Statistical analysis

All experiments were performed at least three times. Statistical analysis was performed by ANOVA, followed by Student-Newman-Keuls tests for experiments involving more than two groups. Student’s t-tests were performed for comparisons between two groups. p-values <0.05 were considered to be statistically significant.

## Results

### Enhanced expression of miR-181a induces hESC decidualization *in vitro*

**Figure 1:**
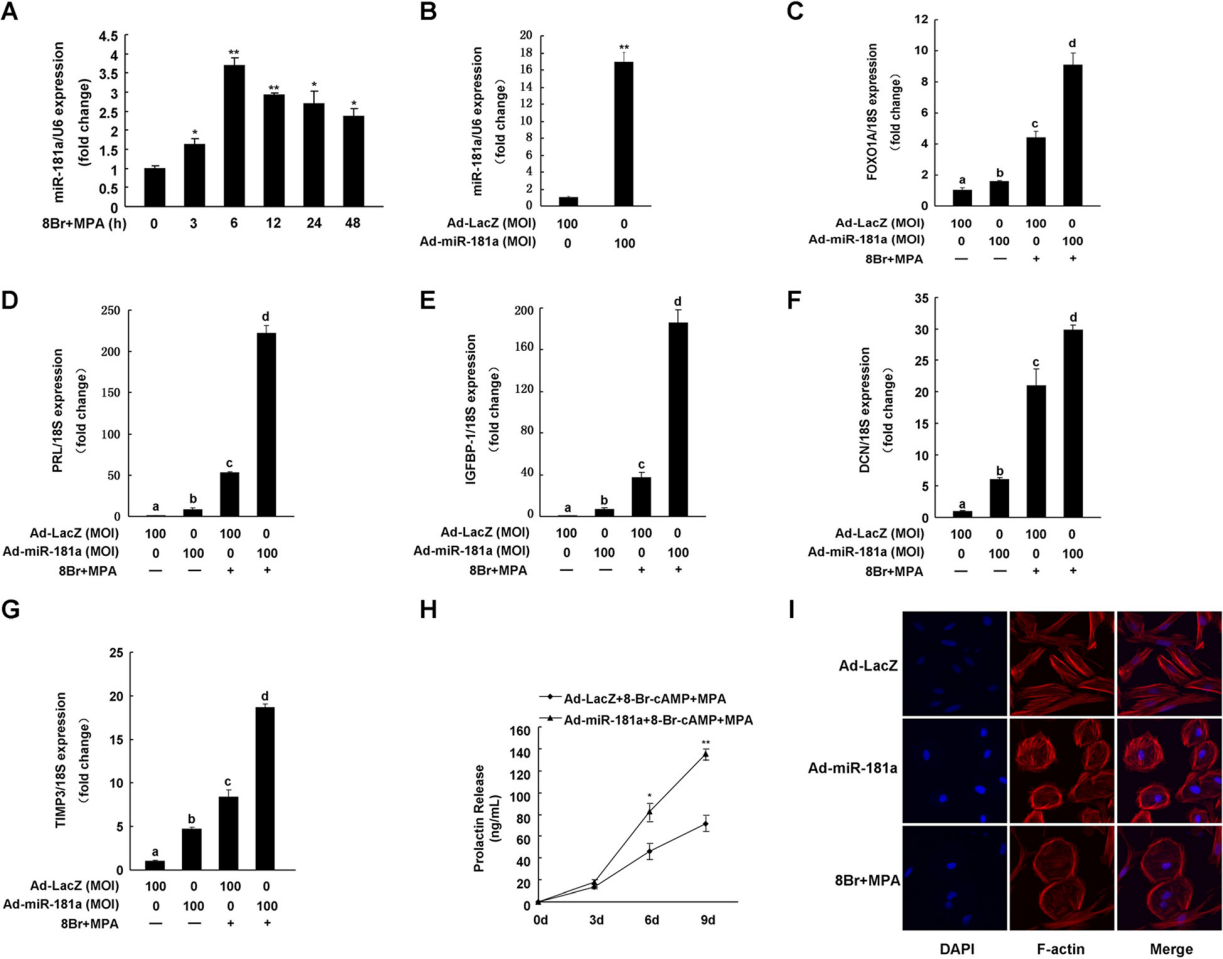
**miR-181a induces hESC decidualization** ***in vitro*** **. (A)** Expression pattern of miR-181a in hESC treated with 0.5 mM 8-Br-cAMP and 1 μM MPA (8Br + MPA) for different periods of time (3, 6, 12, 24, and 48 h, respectively) was evaluated by qRT-PCR. *p < 0.05, **p < 0.01. hESC were infected with Ad-miR-181a or Ad-LacZ (MOI = 100). After 24 h, these cells were treated with 0.5 mM 8-Br-cAMP and 1 μM MPA as indicated for an additional 72 h. miR-181a **(B)**, FOXO1A **(C)**, PRL **(D)**, IGFBP1 **(E)**, DCN **(F)**, and TIMP3 **(G)** mRNA levels were measured by qRT-PCR. **p < 0.01, bars labeled with different letters indicate statistically significant differences (p < 0.05). **(H)** hESC were infected with Ad-miR-181a or Ad-LacZ (MOI = 100) for 24 h followed by treatment with 0.5 mM 8-Br-cAMP and 1 μM MPA for the indicated times. Prolactin released into the medium was detected by ELFA. *p < 0.05, **p < 0.01, compared with Ad-LacZ treated with 8-Br-cAMP and MPA. **(I)** Immunofluorescence using Alexa Fluor 594-conjugated phalloidin to label actin filaments was performed to analyze the morphological transformation of hESC.

miR-181a expression pattern was investigated in hESC treated with 8-Br-cAMP and MPA for different periods of time (3, 6, 12, 24, and 48 h, respectively). qRT-PCR results showed that miR-181a expression was increased after 8-Br-cAMP and MPA treatment, and most efficiently elevated at 6 h time point (Figure 1A). To investigate whether miR-181a regulates hESC decidualization, we overexpressed miR-181a using adenoviral technology (Figure 1B). Then we tested decidualization-related gene expression and observed that adenovirus-mediated overexpression of miR-181a in hESC markedly increased FOXO1A mRNA expression and the expression of its targeted genes (PRL, IGFBP-1, DCN, and TIMP3) (Figure 1C–G). In addition, when 8-Br-cAMP and MPA were added to miR-181a-overexpressing cells, the levels of FOXO1A, PRL, IGFBP-1, DCN, and TIMP3 were higher than those observed with Ad-miR-181a or 8-Br-cAMP and MPA treatment alone (Figure 1C–G). Moreover, miR-181a overexpression significantly increased decidual PRL secretion in a time-dependent manner following the stimulation of decidualization (Figure 1H).

Because the decidualization of hESC is also characterized by the transformation of fibroblast-like hESC into a round, epithelioid shape, we further examined whether miR-181a affects the organization of the F-actin cytoskeleton. As shown in Figure 1I, decidualized hESC treated with 8-Br-cAMP and MPA displayed more polygonal cell morphology with a random distribution of F-actin filaments compared with non-decidualized hESC. In the absence of exogenous hormones, miR-181a overexpression caused the long, fibroblast-like shape of hESC to become noticeably rounder and the actin filaments to rearrange without direction.

### miR-181a inhibition compromises hESC decidualization *in vitro*

**Figure 2:**
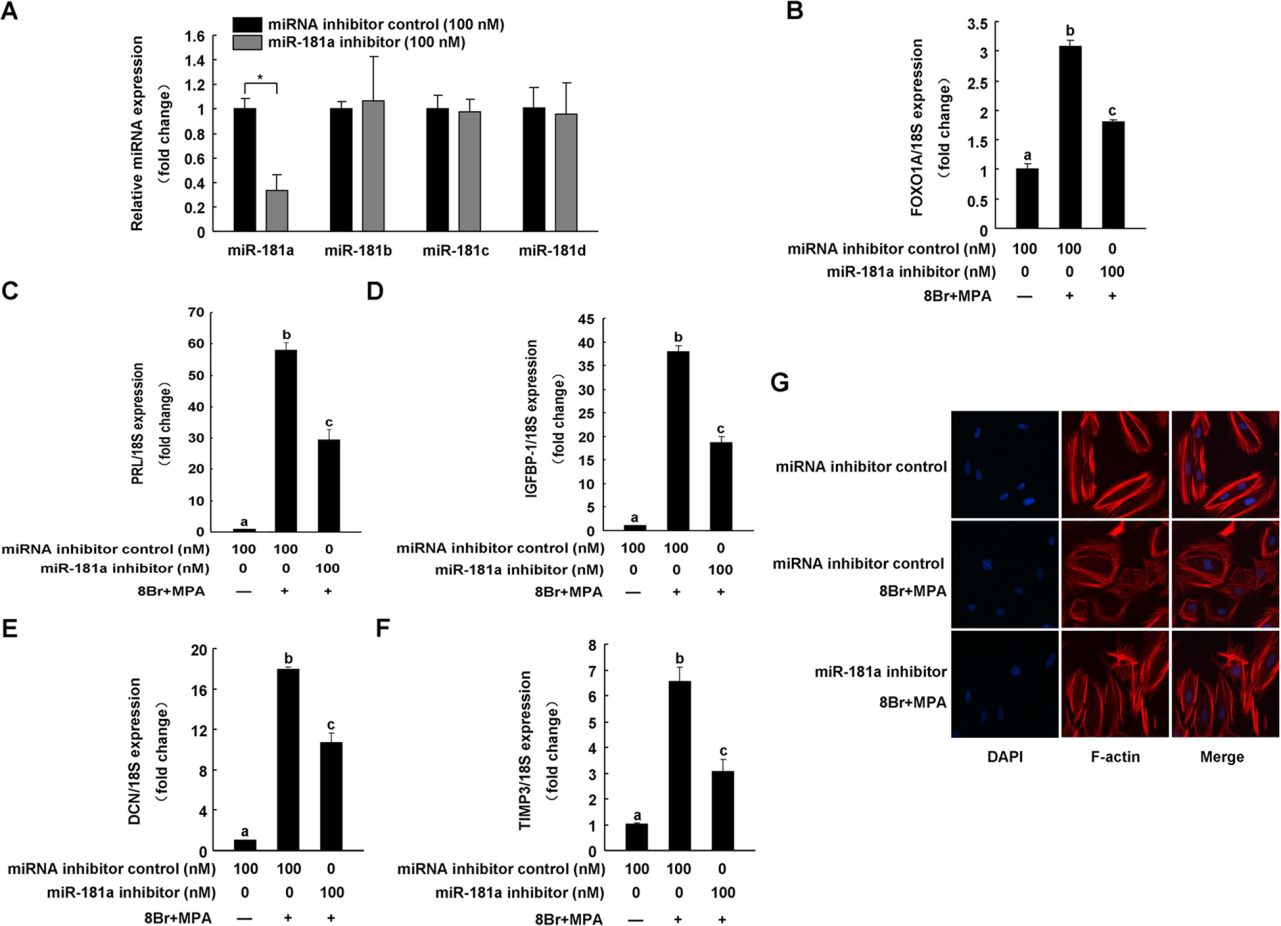
**miR-181a inhibition attenuates hESC decidualization** ***in vitro*** **.** hESC were transfected with a miR-181a inhibitor or a miRNA inhibitor negative control (miRNA inhibitor control, 100 nM) for 24 h, and then cells were treated with or without 0.5 mM 8-Br-cAMP and 1 μM MPA (8Br + MPA) for an additional 72 h. miR-181 family **(A)**, FOXO1A **(B)**, PRL **(C)**, IGFBP1 **(D)**, DCN **(E)**, and TIMP3 **(F)** mRNA expression levels were examined by qRT-PCR. *p < 0.05, bars labeled with different letters indicate statistically significant differences (p < 0.05). **(G)** The decidual transformation change of hESC based on the distribution of the actin filaments.

We transfected hESC with a synthesized anti-sense oligonucleotide of miR-181a (miR-181a inhibitor) to corroborate its regulatory effect on decidualization markers. miR-181a inhibitor specifically suppressed endogenous miR-181a expression without affecting miR-181b, miR-181c, or miR-181d levels in hESC (Figure 2A). Inhibition of miR-181a in hESC led to a significant decrease in FOXO1A, PRL, IGFBP-1, DCN, and TIMP3 gene expression induced by 8-Br-cAMP and MPA (Figure 2B–F). Furthermore, decidualized hESC reverted from a round, epithelioid-like morphology to a fibroblast-like phenotype after miR-181a inhibitor transfection (Figure 2G).

### miR-181a inhibits KLF12 expression

**Figure 3:**
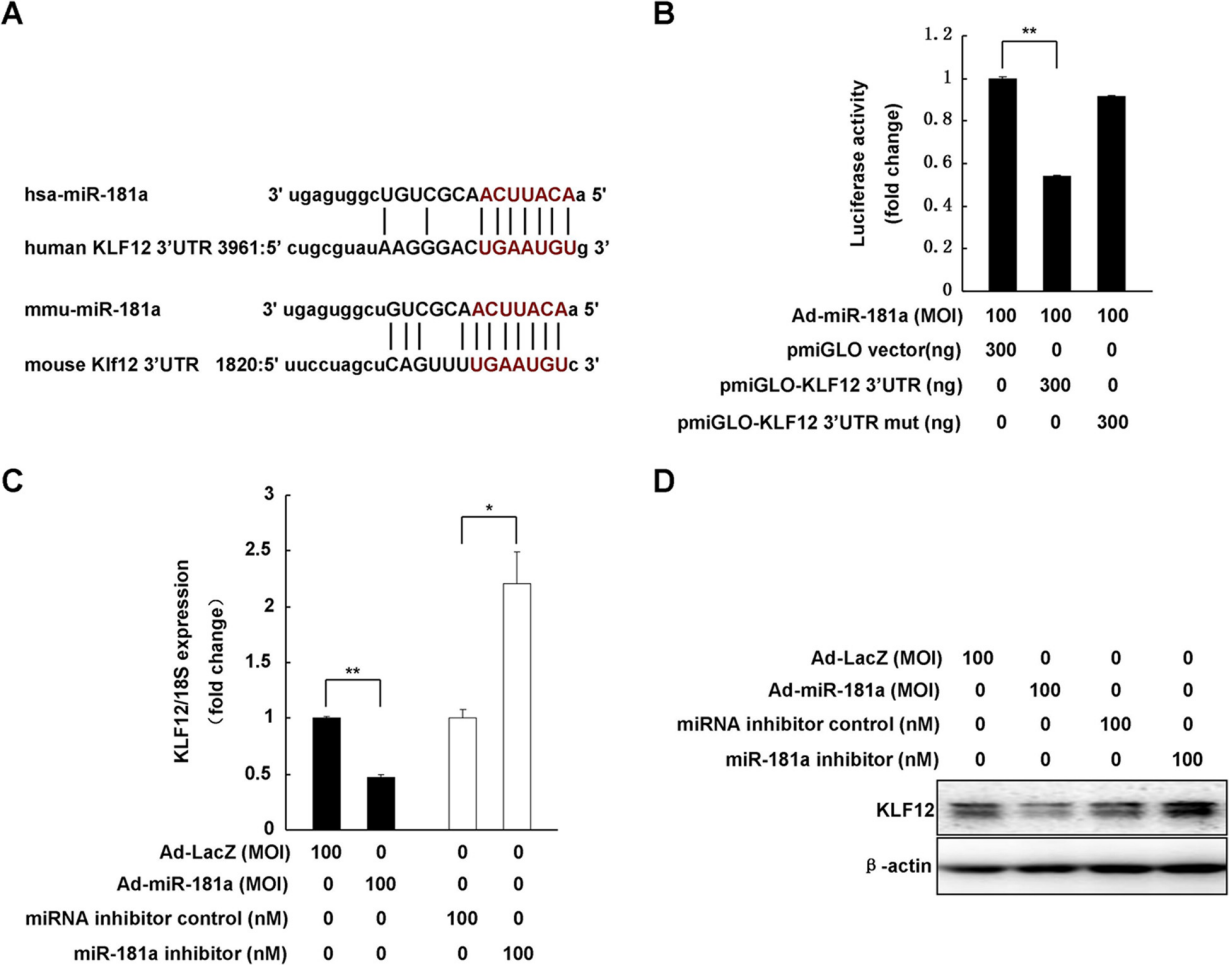
**miR-181a inhibits KLF12 expression. (A)** Putative miR-181a-targeting sites in the human and mouse KLF12 mRNA 3′UTRs. **(B)** Analysis of miR-181a modulation on wild-type or mutant KLF12 3′UTR luciferase reporter plasmids. **p < 0.01 (n = 3). hESC were infected with Ad-miR-181a or transfected with miR-181a inhibitor as indicated for 48 h. qRT-PCR and western blotting were performed to examine endogenous KLF12 mRNA **(C)** and protein **(D)** expression. *p < 0.05, compared with control group, **p < 0.01, compared with the Ad-LacZ group.

We next sought to identify the potential target driving miR-181a-mediated hESC decidualization *in vitro*, and focused on KLF12 gene, which is down-regulated in decidualized hESC [22]. Based on the mouse and human KLF12 mRNA sequences deposited in GenBank (NCBI Reference Sequences: NM_010636.3 and NM_007249.4), we found a miR-181a seed target region within the KLF12 mRNA 3′UTR (Figure 3A). To verify that KLF12 is a potential target of miR-181a, a luciferase-based reporter assay was performed using the KLF12 3′UTR. Increased expression of miR-181a in hESC significantly decreased luciferase reporter activity by approximately 50% (Figure 3B). A mutated KLF12 3′UTR in which seven nucleotides of the miR-181a binding site were abolished no longer responded to miR-181a modulation (Figure 3B).

Consistent with KLF12 3′UTR reporter results, miR-181a overexpression in hESC significantly down-regulated endogenous levels of KLF12 at both the mRNA and protein levels. Reduced miR-181a expression resulted in the up-regulation of KLF12 mRNA and protein expression in hESC (Figure 3C and D).

### KLF12 overexpression attenuates miR-181a-mediated decidualization events

**Figure 4:**
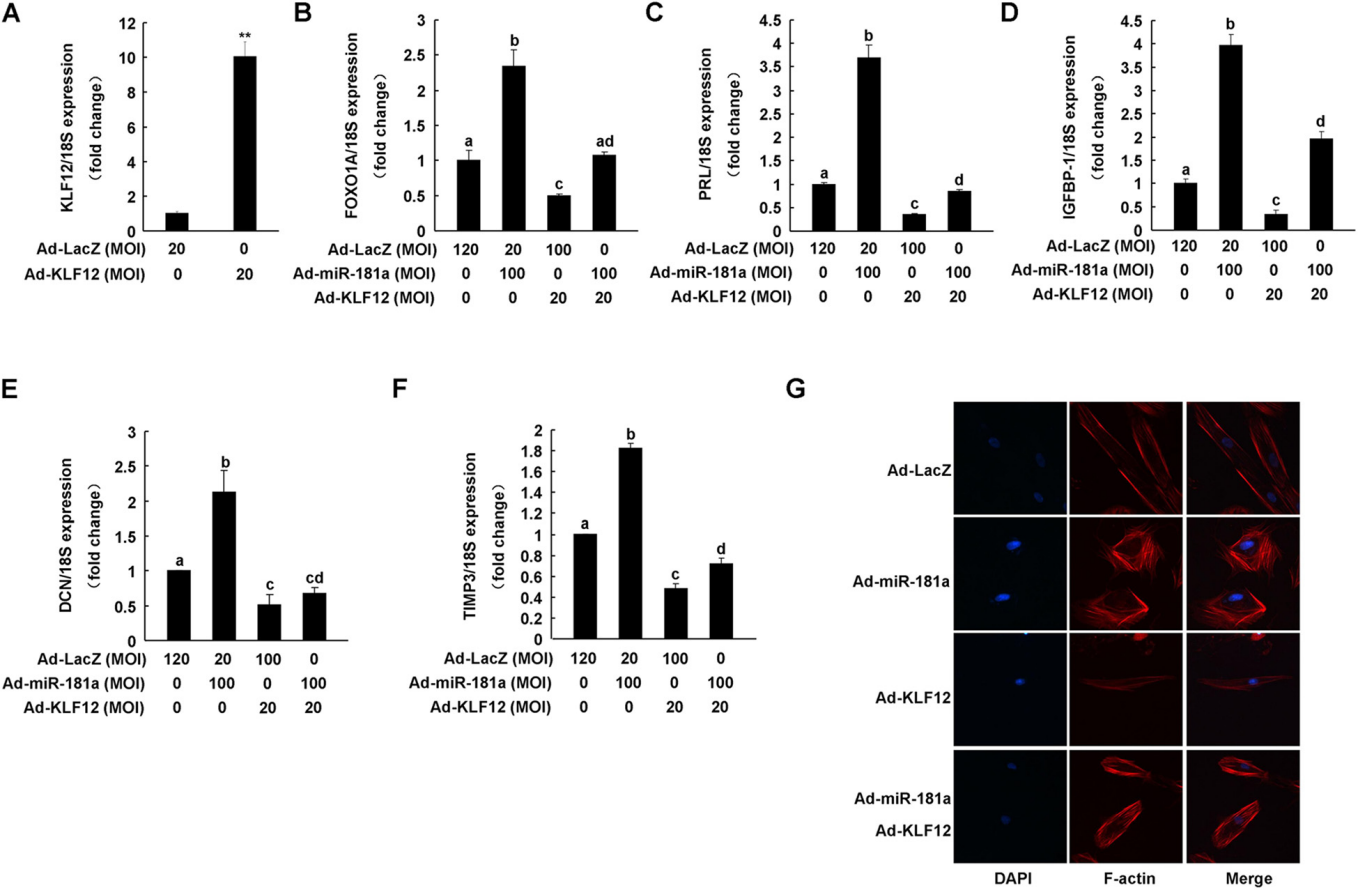
**KLF12 overexpression represses the miR-181a-induced morphological and biochemical transformation of hESC.** hESC were infected with Ad-miR-181a (100 MOI) and/or Ad-KLF12 (20 MOI) as indicated for 72 h. KLF12 **(A)**, FOXO1A **(B)**, PRL **(C)**, IGFBP1 **(D)**, DCN **(E)**, and TIMP3 **(F)** mRNA levels were measured by qRT-PCR. **p < 0.01, bars labeled with different letters indicate statistically significant differences (p < 0.05). **(G)** The morphological changes of hESC were detected by immunofluorescence staining.

Finally, we assessed whether KLF12 is involved in the process of miR-181a-induced hESC decidualization. Adenovirus-mediated KLF12 overexpression (Figure 4A) in hESC suppressed the mRNA expression of FOXO1A, PRL, IGFBP-1, DCN, and TIMP3, compared to Ad-LacZ group (Figure 4B–F). KLF12 overexpression also attenuated miR-181a-enhanced mRNA expression of these genes (Figure 4B–F). Moreover, KLF12 overexpression blocked miR-181a-induced epithelioid-like morphological changes of hESC (Figure 4G).

## Discussion

The transformation of endometrium into decidua is essential for normal implantation of the blastocyst, a process in which many key proteins and growth factors play fundamental roles [2,3]. The participation of estrogen and progesterone is vital for stromal cell decidualization, as progesterone receptor or estrogen receptor knockout mice both fail to display endometrial decidualization and as 8-Br-cAMP and MPA treatment can induce hESC decidualization *in vitro* [22,25,26]. miRNAs are also involved in this process, although their exact role in normal embryonic formation, endometrial preparation for pregnancy, and decidualization remains unclear [11,13,15]. Here, we found that miR-181a level is increased in the process of 8-Br-cAMP and MPA-induced hESC decidualization *in vitro*, suggesting that miR-181a may play functions in this process.

miR-181a has been demonstrated to be a key modulator of cellular differentiation, including hematopoietic lineages and myoblasts, as well as T-cell sensitivity and selection [27-29]. Recently, we identified that miR-181a suppresses mouse granulosa cell proliferation by targeting activin receptor IIA (acvr2a) and thus regulates activin-induced gene expression [24]. In this study, our data confirmed that miR-181a promotes decidualization-related gene expression and causes a noticeable change in stromal cell shape. Furthermore, miR-181a inhibition causes an impaired induction of the decidual reaction by 8-Br-cAMP and MPA. These results demonstrate that miR-181a plays a positive role in hESC decidualization. Other recent studies support the crucial roles that miRNAs play in decidualization. For example, Dicer mRNA and protein levels were significantly up-regulated after decidualization treatment using cAMP and MPA [11]. miR-222 regulates hESC differentiation by targeting CDKN1C/p57kip2 expression [30]. miR-135b also targets HOXA10, which is essential for female fertility and decidualization [31,32]. Moreover, miR-141, miR-143, and miR-193 are differently expressed in the mouse uteri before and after embryo implantation [33-35]. miR-181a is also reported to be highly expressed in the porcine endometrium on day 15 gestation, compared to that on day 26 and 50 gestation [15]. However, the function of miR-181a in endometrial decidualization is unclear. Our study confirms that miR-181a induces hESC decidualization.

The effects of progesterone are mediated through interactions with the progesterone receptor (PGR). PGR physically associates with other nuclear transcription factors, such as FOXO1A and the estrogen receptor, to regulate decidualization-specific gene expression [36,37]. The transcription factor FOXO1A is critical for decidualization and promotes the expression of decidualization-associated targeted genes, such as PRL, IGFBP-1, DCN, and TIMP3 during the decidualization process [38,39]. The transcriptional ability of FOXO1A is regulated by many factors in the process of endometrial decidualization, such as PI3K/Akt, PGR, and HoxA10 [40-42]. To date, the function of miRNAs involved in regulating FOXO1A expression and activation is largely uncovered. In this study, we revealed that overexpression of miR-181a increases FOXO1A mRNA expression and miR-181a inhibitor suppresses 8-Br-cAMP and MPA-induced FOXO1A expression, indicating that miR-181a mediates the promoting effect of decidual stimuli on FOXO1A expression.

To identify physiological targets of miR-181a involved in the process of miR-181a-induced decidual gene expression, we focused on KLF12, a novel transcription factor identified by our laboratory that negatively regulates hESC decidualization [22]. A luciferase assay demonstrated that miR-181a interacts with the 3′UTR of KLF12 and down-regulates KLF12 at the transcriptional and translational levels. Re-expression of KLF12 abolished miR-181a-induced decidualization, suggesting that KLF12 is a critical mediator of miR-181a-induced decidualization. Members of the KLF family of zinc-finger transcription factors are critical for the development of uterine receptivity and the differentiation of stromal cells [19,20]. KLF12 protein is significantly decreased after the stimulation of decidualization, and KLF12 overexpression in hESC significantly represses the expression of decidualization marker genes and cell morphology changes [22]. Interestingly, based on the human FOXO1A promoter sequence (accession no: 11424), the dPRL promoter sequence (accession no: 37139), and the IGFBP-1 promoter sequence (accession no: 37680) deposited in the Transcriptional Regulatory Element Database [43], we found conserved CAGTGGG elements within the promoter core regions of these genes, suggesting the possibility of a direct role for KLF12 in regulating their expression. In the future, we will further study the molecular mechanisms of miR-181a and KLF12 in decidualization.

Aberrant miRNA expression is associated with a wide variety of human diseases. Endometrial miR-181a and miR-98 are aberrantly expressed in endometrial tumors [44], and miR-181a plays a critical role in epithelial ovarian cancer (EOC) progression through the regulation of the epithelial-mesenchymal transition by modulating the TGF-β signaling pathway [45]. We recently found that KLF12 was markedly up-regulated in endometrium from endometriosis and RIF patients (unpublished data), and we will further investigate the expression patterns of miR-181a in these patients to better understand the role miR-181a plays in the pathogenesis of these diseases.

## Conclusions

Together, this study highlights a novel role of miR-181a and KLF12 in the decidualization process of human endometrial stromal cell. Our findings provide novel potential biomarkers and therapeutic targets for diseases associated with defective decidualization.

## Abbreviations

acvr2a: Activin receptor IIA
CREB1/3: cAMP response element binding protein 1/3
DCN: Decorin
Esr1: Estrogen receptor 1
ETS1: v-ets erythroblastosis virus E26 oncogene homolog 1
FOXO1A: Forkhead headbox O1A
hESC: Human endometrial stromal cell
HOXA10: Homeobox A10
IGFBP-1: Insulin-like growth factor binding protein-1
IL-1: Interleukin-1
KLF12: Krüppel-like factor 12
miR-181a: microRNA-181a
MPA: Medroxyprogesterone acetate
PR: Progesterone receptor
PRL: Prolactin
RIF: Repeated implantation failure
TNF-*α*: Tumor necrosis factor-α
3′UTR: 3′ untranslated region
8-Br-cAMP: 8-bromoadenosine-cAMP
